# The description of two new species of *Chloromyxum* from skates in the Argentine Sea reveals that a limited geographic host distribution causes phylogenetic lineage separation of myxozoans in Chondrichthyes

**DOI:** 10.1051/parasite/2018051

**Published:** 2018-09-12

**Authors:** Delfina María Paula Cantatore, Manuel Marcial Irigoitia, Astrid Sibylle Holzer, Pavla Bartošová-Sojková, Hana Pecková, Ivan Fiala, Juan Tomás Timi

**Affiliations:** 1 Laboratorio de Ictioparasitología, Instituto de Investigaciones Marinas y Costeras (IIMyC), Consejo Nacional de Investigaciones Científicas y Técnicas (CONICET), Universidad Nacional de Mar del Plata Mar del Plata Argentina; 2 Institute of Parasitology, Biology Centre of the Czech Academy of Sciences Branišovská 31 37005 České Budějovice Czech Republic

**Keywords:** *Chloromyxum* sp., SSU rDNA sequences, elasmobranchs, *Atlantoraja castelnaui*, *Zearaja chilensis*, *Rioraja agassizii*

## Abstract

During a survey on the myxosporean fauna of Rajiformes from the Atlantic coast of Argentina, in waters off Buenos Aires Province (34°–42°S; 53°–62°W), the gall bladders of 217 specimens belonging to seven species of skates, representatives of two families, were examined. As a result, three species of *Chloromyxum* Mingazzini, 1890, namely *C. atlantoraji* n. sp., *C. zearaji* n. sp. and *C. riorajum* Azevedo, Casal, Garcia, Matos, Teles-Grilo and Matos, 2009 were found infecting three endemic host species, the spotback skate *Atlantoraja castelnaui* (Arhynchobatidae), the yellownose skate *Zearaja chilensis* (Rajidae) and the Rio skate *Rioraja agassizii* (Arhynchobatidae), respectively. These species were described based on myxospore morphology and morphometry characterization, as well as by providing their small subunit ribosomal DNA (SSU rDNA) sequences. The SSU rDNA-based phylogenetic analyses showed that these three species constituted a well-established monophyletic subclade within the marine *Chloromyxum* clade, while branches subtending the other *Chloromyxum* species were poorly resolved or unresolved, independently of the host taxonomic identities (Carchariniformes, Myliobatiformes, Orectolobiformes, Pristiophoriformes, Rajiformes, Squaliformes and Torpediniformes) and/or host geographic distribution (Atlantic coast of Portugal, Atlantic coast of the USA, Australian waters or Mediterranean Sea). The possible causes of these discrepancies are discussed, providing new insights into the phylogeny of the marine *Chloromyxum* clade.

## Abstract (fr)

Lors d’une étude de la faune des Myxozoaires des Rajiformes de la côte atlantique argentine, dans les eaux situées au large de la province de Buenos Aires (34°–42°S; 53°–62°O), les vésicules biliaires de 217 spécimens appartenant à sept espèces, représentants deux familles, ont été examinés. En conséquence, trois espèces de *Chloromyxum* Mingazzini, 1890, à savoir *C. atlantoraji* n. sp., *C. zearaji* n. sp. et *C. riorajum* Azevedo, Casal, Garcia, Matos, Teles-Grilo et Matos, 2009 ont été trouvées, infectant trois espèces hôtes endémiques, *Atlantoraja castelnaui* (Arhynchobatidae), *Zearaja chilensis* (Rajidae) et *Rioraja agassizii* (Arhynchobatidae), respectivement. Ces espèces sont décrites sur la base de la morphologie et de la morphométrie des myxospores, ainsi qu’en fournissant leurs petites séquences d’ADN ribosomal (SSU ADNr). Les analyses phylogénétiques basées sur l’ADNr SSU ont montré que ces trois espèces constituaient un sous-clade monophylétique bien établi dans le clade des *Chloromyxum* marins, tandis que les branches sous-jacentes aux autres espèces de *Chloromyxum* étaient mal ou non résolues, indépendamment des identités taxonomiques hôtes (Carchariniformes, Myliobatiformes, Orectolobiformes, Pristiophoriformes, Rajiformes, Squaliformes et Torpediniformes) et/ou de la répartition géographique de l’hôte (côte atlantique du Portugal, côte atlantique des États-Unis, eaux australiennes ou mer Méditerranée). Les causes possibles de ces divergences sont discutées, fournissant de nouvelles informations sur la phylogénie du clade des *Chloromyxum* marins.

## Introduction

Along the Southwestern Atlantic Shelf, south of 34°S, 106 elasmobranch species have been reported among permanent and occasional inhabitants, two of which are holocephalans, 55 are sharks, and 49 are batoids [20, 22, 60, 62, 75]. Elasmobranch richness is heterogeneous across this region, mainly associated with a marked temperature gradient [45, 59, 63], with the presence of marine fronts [58] and with paleogeographical processes [32], with the maximum richness observed at the ecotonal region ranging between 34° and 42°S in waters off Buenos Aires Province [20]. This region harbours more than 30 species of Rajiformes belonging to seven genera, four of which are endemic [32], constituting an area with a high degree of endemism and great diversity at global scale. Nonetheless, the information available about the parasites of elasmobranchs inhabiting the Argentine Sea is fragmentary. Particularly, the diversity of myxozoans in elasmobranch from this region has not been widely studied and only five species have been described, all belonging to the genus *Chloromyxum* Mingazzini, 1890 [64], namely: *C. ovatum* Jameson, 1929, *C. liae* Kuznetsova, 1977, *C. transversocostatum* Kuznetsova, 1977, *C. multicostatum* Kuznetsova, 1977 and *C. parvicostatum* Kuznetsova, 1977 [27, 44].

*Chloromyxum* is the fourth largest myxozoan genus (Cnidaria: Myxozoa) comprising about 140 nominal species [25, 72] commonly infecting the gall bladder of freshwater and marine fishes. In elasmobranchs, 25 *Chloromyxum* spp. with a worldwide distribution have been described so far see [35, 72]. *Chloromyxum leydigi* Mingazzini, 1890 [64], the type species, was originally described, without specifying the type host, from the gall bladder of numerous elasmobranch species ranked to different genera, namely *Mustelus* Fischer von Waldheim, *Galeus* Cuvier, *Raja* Linnaeus, *Scyllium* Cuvier, *Squatina* Duméril, *Torpedo* Houttuyn and *Trygon* Cuvier [34, 44, 53, 56, 57, 64, 69, 73]. This suggests that *C. leydigi* is in fact an assemblage of several species [6, 35, 73], a phenomenon previously shown for other chloromyxids [12]. Nonetheless, *C. leydigi* gained specific identity through its morphological and ultrastructural redescription, and sequencing of the small subunit ribosomal DNA (SSU rDNA) from two different host species [28, 30, 73]. In the last study, the type host (*Torpedo marmorata* Risso) and the type locality (Mediterranean Sea) of the parasite were assigned [73]. Previous studies have shown that *Chloromyxum* is a polyphyletic genus [28, 30], with its type species clustering with other myxozoans infecting the gall bladder of marine elasmobranchs to form the marine *Chloromyxum* clade [47], also named *Chloromyxum sensu stricto* clade [35], placed at the basis of the freshwater myxosporean lineages [28, 29, 31].

The present study provides the morphological and molecular description of two new species of *Chloromyxum* based on material collected from the gall bladder of two species of Rajiformes captured on the Atlantic coast of Argentina: the spotback skate *Atlantoraja castelnaui* (Miranda Ribeiro) (Arhynchobatidae) and the yellownose skate *Zearaja chilensis* (Guichenot) (Rajidae). An additional locality is also reported for *C. riorajum* Azevedo, Casal, Garcia, Matos, Teles-Grilo and Matos, 2009 collected from its type host, the Rio skate *Rioraja agassizii* (Müller and Henle) (Arhynchobatidae) [8]. Analyses of their SSU rDNA sequences allowed us to evaluate their systematic relationships and phylogenetic position amongst other chloromyxids parasitising elasmobranchs within the marine *Chloromyxum* clade, emphasising the interpretation in relation to host identity and host geographic distribution.

## Materials and methods

### Host and parasite collection and processing

The gall bladders of a total of 217 skates belonging to 7 species were examined for myxosporean infections, including 22 specimens of the southern thorny skate *Amblyraja doellojuradoi* (Pozzi) (Rajidae), 14 specimens of *A. castelnaui*, 27 specimens of the white-dotted skate *Bathyraja albomaculata* (Norman) (Arhynchobatidae), 18 specimens of *R. agassizii*, 66 specimens of the bignose fanskate *Sympterygia acuta* Garman (Arhynchobatidae), 48 specimens of the smallnose skate *S. bonapartii* Müller and Henle (Arhynchobatidae), and 22 specimens of *Z. chilensis*, all obtained from commercial trawlers in waters off Buenos Aires Province (34°–42°S; 53°–62°W), Argentina during 2014.

Fish were kept fresh on ice until examination. Upon necropsy, the livers were removed entirely and the bile from the gall bladders was extracted by a sterile syringe. A drop of bile of each gall bladder was then placed on a microscope slide and examined using the Leica DM2500 stereoscopic microscope for the detection of myxosporeans. The rest of the bile of the parasitized fishes was then processed for observation by light microscopy (LM), scanning electron microscopy (SEM), and for molecular characterization.

Valid names of host species for previously recorded myxosporeans are in accordance with the Catalog of Fishes [26].

### Morphological analysis of myxospores by light microscopy

Digital photographs of fresh myxospores observed under the differential interference contrast (DIC) were taken using the Leica DM2500 stereoscopic microscope equipped with the Leica DFC295 camera and then measured in ImageJ v.1.45 s [76]. Measurements of myxospores followed the guidelines proposed by Lom and Arthur [55] for species descriptions of Myxosporea. All measurements are given in micrometres as a mean ± standard deviation (*SD*), followed by range in parentheses and the number of myxospores measured for each myxospore dimension.

### Morphological analysis of myxospores by SEM

The gall bladder contents of each host species showing high intensity of myxosporean infections were fixed in 2.5% (v/v) glutaraldehyde buffered in 0.2 M sodium cacodylate (pH 7.2) for 24 h at 4 °C, and then rinsed in the same buffer (2 × 30 min) at the same temperature. The samples were then dehydrated in an ascending ethanol series, transferred into 50% hexamethyldisilazane (HMDS) in 100% ethanol followed by two changes of 100% HMDS each for 45 min (partially modified from Rocha and Azevedo [71]). A drop of sample was then put onto a coverslip, the excess of HMDS was allowed to air dry, and then the sample was coated with gold-palladium and observed and photographed with the JEOL JSM 6460-LV scanning electron microscope (JEOL, Tokyo, Japan).

### Morphometric comparison of myxospores

Morphometric differences among *Chloromyxum* myxospores from different skate species were explored using the canonical analysis of principal coordinates (CAP) [3, 4]. Potential over-parameterisation was prevented by choosing the number of principal coordinates ordination (PCO) axes (m) that maximised a leave-one-out allocation success to groups [2]. CAP analyses were based on Euclidean distances on the variables myxospore thickness, length and width, and polar capsule length and width.

In addition to CAP analysis, differences in dimensions of *Chloromyxum* myxospores from different host species were tested using the one-way distance-based permutational multivariate analysis of variance (PERMANOVA) [1, 4]. PERMANOVA analysis was conducted over 9999 permutations under the default permutation method (permutation of residuals under a reduced model) and sum of squares type III (partial) [4]. When differences were detected by PERMANOVA, pairwise comparisons were used to determine which samples differed. Since PERMANOVA is sensitive to differences in multivariate dispersion among samples (*sensu* homogeneity of variances, which can inflate Type 1 error even when centroids have identical locations), differences in dispersion were tested using the routine PERMDISP [4]. Dispersions were measured as distances to the centroids and each term in the analysis was tested using 9999 permutations.

All multivariate statistical procedures on morphometric data were performed by PRIMER v.6. [18, 19] and PERMANOVA + for PRIMER [4].

### DNA extraction, PCR amplification, cloning and sequencing

Selected positive samples were fixed and preserved in 96% ethanol at 4 °C for molecular characterization by sequencing of SSU rDNA. Each sample was pelleted twice at 3000 rpm for 5 min and washed with DNAse-free water, and the ethanol was removed. Then samples were stored in 400 μL TNES urea buffer (10 mM Tris-HCl with pH 8, 125 mM NaCl, 10 mM EDTA, 0.5% SDS and 4 M urea) [5] for subsequent DNA extraction. Total DNA was extracted by a simplified phenol-chloroform extraction protocol [41], after an overnight digestion with proteinase K (50 μg mL^−1^) at 55 °C. The extracted DNA was re-suspended in 50 μL of sterile distilled water and left to dissolve at 4 °C until further use.

Almost complete SSU rDNA sequences of *Chloromyxum* species from *A. castelnaui* as well as from *Z. chilensis*, and partial SSU rDNA sequences of *Chloromyxum* species infecting *R. agassizii* were obtained by assembling overlapping parts amplified with both universal and myxozoan-specific primers. Thereby, the reaction with the universal eukaryotic ERIB1 (5′-ACCTGGTTGATCCTGCCAG-3′) and ERIB10 (5′-CTTCCGCAGGTTCACCTACGG-3′) primers (both [11]) in the first PCR was followed by nested PCR with MyxGP2F (5′-TGGATAACCGTGGGAAA-3′ [49]) and ACT1r (5′-AATTTCACCTCTCGCTGCCA-3′ [36]) for all *Chloromyxum* species, and by ERIB1 + ACT1r and by MyxospecF (5′-TTCTGCCCTATCAACTWGTTG-3′) + MyxospecR (5′-GGTTTCNCDGRGGGMCCAAC-3′) (both [28]) for *Chloromyxum* species from *A. castelnaui* and *Z. chilensis*. Additionally, for the *Chloromyxum* species from *A. castelnaui* and *Z. chilensis*, an overlapping part was also amplified with MyxospecF + ERIB10.

Polymerase chain reactions (PCRs) of the SSU rDNA were performed in a 25 μL reaction using 1 × Taq buffer, 250 μM of each dNTPs, 10 pmol of each primer, 1 U of Taq-Purple polymerase (Top-Bio, Czech Republic), 1 μL of DNA and sterile distilled water. PCR cycling parameters used for the amplification of SSU rDNA sequences for the primary/nested PCR were as follows: denaturation at 95 °C for 3 min, then 30 cycles of amplification at 95 °C for 1 min, 48 °C/50 °C for 1 min, 72 °C for 1 or 2 min and followed by a 10 min extension at 72 °C. All amplified products were purified using Gel/PCR DNA Fragments Extraction Kit (Geneaid Biotech Ltd., USA).

Problematic amplicons with low DNA concentration for the amplified segment MyxospecF-ERIB10 of *Chloromyxum* species infecting *A. castelnaui* were cloned into the pDrive Vector with a Qiagen PCR Cloning Kit (Qiagen, Germany) and transformed into XL-1 *E. coli* strain competent cells. Positive colonies were PCR screened with M13 forward and reverse primers. Plasmid DNA was isolated using a High Pure Plasmid Isolation Kit (Roche Applied Science, Germany) and two colonies were sequenced. The PCR products and plasmids were commercially Illumina sequenced (https://www.seqme.eu).

### Phylogenetic analyses

The overlapping partial sequences of SSU rDNA were assembled in SeqMan II v5.05 (DNASTAR Inc., Madison, Wisconsin, USA). New sequences were deposited in GenBank with accession numbers MG652633, MG652632 and MG652631. The SSU rDNA alignment was created in the MAFFT v7.017 [48] using E-INS-i strategy and default parameters (gap opening penalty: 1.53 and gap extension penalty 0.0). The alignment contained newly obtained sequences and all sequences of taxa of the elasmobranch-infecting *Chloromyxum* clade see [35, 72] retrieved from GenBank. *Chloromyxum cristatum* Léger, 1906 (GenBank accession no: AY604198) and *Myxidium lieberkuehni* Bütschli, 1882 (GenBank accession no: X76639) were set as the outgroup. Ambiguously aligned regions of the alignment were eliminated and the ends of the alignment were trimmed.

Phylogenetic analyses were performed using maximum likelihood (ML), maximum parsimony (MP) and Bayesian inference (BI). ML was done in the RAxML v7.0.3. [77] with GTR + Γ model of evolution. MP was performed in the PAUP* v4.0b10 [78] using a heuristic search with random taxa addition and the tree bisection reconnection (TBR) swapping algorithm. All characters were treated as unordered, the *T*_s_:*T*_v_ ratio was set to 1:2 and gaps were treated as missing data. For ML and MP, clade supports were assessed with bootstrapping of 1000 replicates with random sequences additions. BI was conducted in MrBayes v3.0 [74] using the GTR + Γ model of evolution. Posterior probabilities were estimated over 1 000 000 generations via two independent runs of four simultaneous Markov chain Monte Carlo chains with every 100th tree saved. Tracer v1.4.1 [70] was used to set the length of the burn-in period. Uncorrected *p*-distance were calculated with default parameters from the alignment using PAUP* v4.0b10.

## Results

Plasmodia and mature myxospores of coelozoic myxozoans consistent with the morphological diagnosis of the genus *Chloromyxum* were observed floating freely in the bile of *A. castelnaui*, *R. agassizii* and *Z. chilensis*. No myxozoan infections were found in the gall bladder of the other four skates examined (*A. doellojuradoi*, *B. albomaculata*, *S. acuta* and *S. bonapartii*).

Morphological and molecular analysis of positive samples revealed the presence of two new species parasitizing *A. castelnaui* and *Z. chilensis*, respectively, and identified the third species, found in *R. agassizii*, as *C. riorajum* [8]. No macroscopic signs of infection were observed.

### Taxonomic position

Phylum Cnidaria Hatschek, 1888

Subphylum Myxozoa Grassé, 1970

Class Myxosporea Bütschli, 1881

Order Bivalvulida Schulman, 1959

Suborder Variisporina Lom and Noble, 1984

Family Chloromyxidae Thélohan, 1892

Genus *Chloromyxum* Mingazzini, 1890

### *Chloromyxum atlantoraji* n. sp.

urn:lsid:zoobank.org:act:65034C3F-2EC2-4A57-AA8B-452DB8072337

*Type host*: the spotback skate, *Atlantoraja castelnaui* (Miranda Ribeiro) (Rajiformes: Arhynchobatidae).

*Type locality*: off the coast of Buenos Aires Province (34°–42°S; 53°–62°W), Argentina.

*Localization of sporogonic stages*: Plasmodia and myxospores floating in the bile.

*Prevalence of infection*: 35.7% (5 infected of 14 examined fishes).

*Type material*: syntype MLP-Oi 4150 (coverslip with gold-palladium coated sample for SEM) and voucher MLP-ZI-RG 417 (bile containing myxospores in absolute ethanol) deposited in the Other Invertebrates Collection and Genetic Resources Collection, respectively, Museo de La Plata, La Plata, Argentina.

*DNA sequences.* SSU rDNA sequence available in GenBank under the accession number MG652633.

*Etymology*: specific epithet refers to generic name of its host.

**Figure 1.**
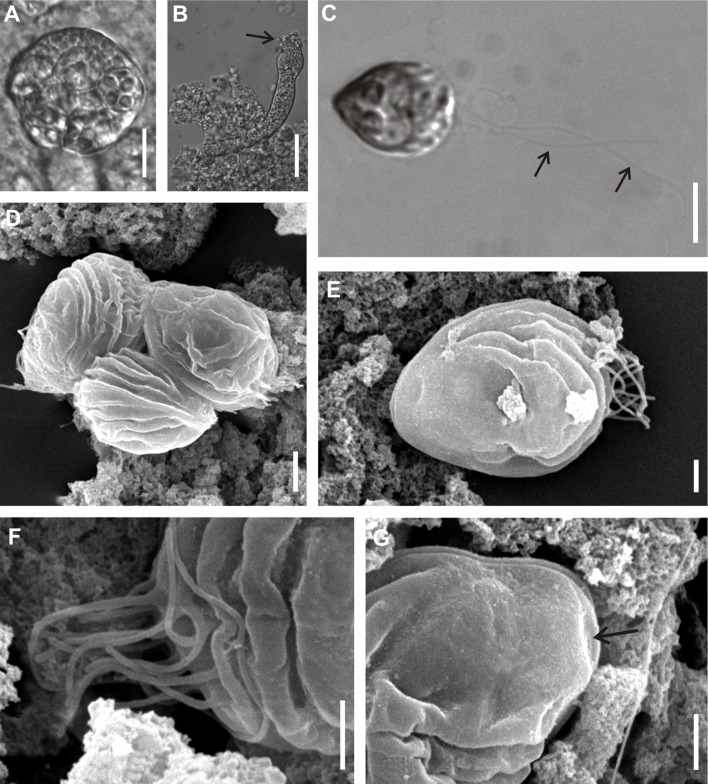
*Chloromyxum atlantoraji* n. sp. from the gall bladder of the spotback skate, *Atlantoraja castelnaui* (Miranda Ribeiro) (Arhynchobatidae, Rajiformes). (A) Spherical polysporic plasmodium (DIC micrographs). (B) Plasmodium displaying highly irregular shape morphology with the presence of peripheral projections (arrow) (DIC micrographs). (C) Mature myxospore showing caudal filamentous projections (arrows), valvular view (DIC micrographs). (D) Mature myxospores, sutural and medial views (SEM). (E) Mature myxospore, valvular view (SEM). (F) Detail of the caudal bundle of filamentous projections (SEM). (G) Detail of the anterior apex showing the mild depression area for polar filaments extrusion (arrow) (SEM). Scale bars: A = 10 μm; B = 20 μm; C = 5 μm; D, F, G = 1 μm; E = 2 μm

**Figure 2.**
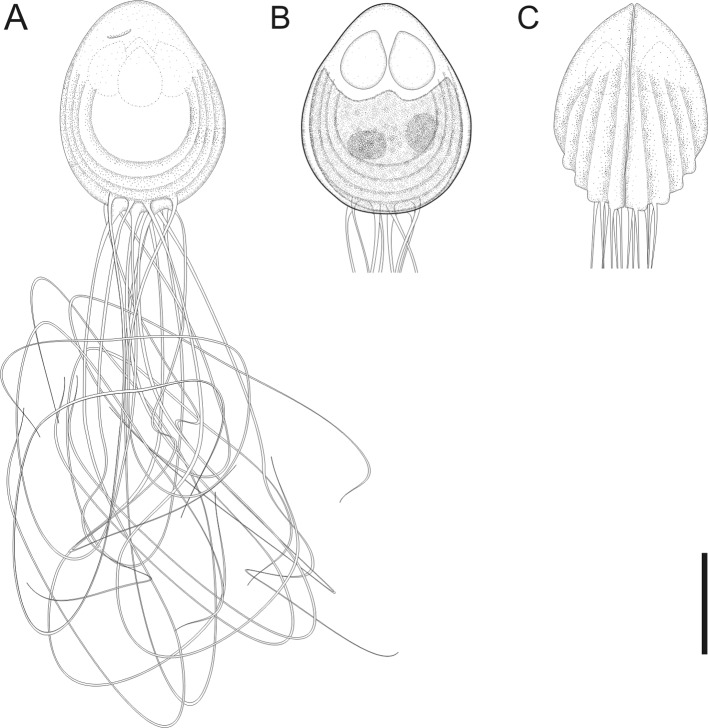
Schematic drawing a myxospore of *Chloromyxum atlantoraji* n. sp. from the gall bladder of the spotback skate, *Atlantoraja castelnaui* (Miranda Ribeiro) (Arhynchobatidae, Rajiformes). (A) Valvular view of a mature myxospore. (B) Valvular view of a mature spore showing a single sporoplasm bearing two nuclei. (C) Sutural view of a mature myxospore, caudal filamentous projections not fully drawn. Scale bars: A, B, C = 5 μm.

*Description* (Figs. 1 and 2)

*Sporogonic stages*: plasmodia polysporic, highly polymorphic; spherical, oval or very irregular in shape. Dimensions varying greatly as a result of polymorphic nature of these structures.

*Myxospores*: mature myxospores ovoid with pointed anterior apex; 10.5 ± 0.4 (9.7–11.4, *n* = 23) long in sutural view, 8.5 ± 0.4 wide (7.7–9.4, *n* = 23) and 8.8 ± 0.6 thick (8.0–9.4, *n* = 23). Myxospore wall composed of two equally sized valves adhering to each other along a slightly sinuous suture line. Each valve with 3–4 elevated surface ridges parallel to suture line and nearly parallel to each other, not reaching the apical pole. A bundle of long caudal filamentous projections, 3 or 4 times longer than the myxospore length, originating at the distal end of shell valves. Four anteriorly pointed, pyriform, equally-sized polar capsules, 3.5 ± 0.3 (3.0–4.5, *n* = 23) in length and 2.4 ± 0.2 (1.9–3.0, *n* = 23) in width. Number of polar filament coils not determined. Extrusion polar filament area located in a mild depression near anterior apex of each shell valve. Single sporoplasm irregular in shape, bearing two nuclei randomly positioned in its matrix.

#### Taxonomic affinities

Among the *Chloromyxum sensu stricto* representatives and taking into account myxospore dimensions (considering the minimum and maximum values of myxospore length, thickness and width provided in the literature), *C. atlantoraji* n. sp. most closely resembles *C. kuhlii* Gleeson and Adlard, 2012, *C. lesteri* Gleeson and Adlard, 2012, *C. leydigi* Mingazzini, 1890, *C. mingazzinii* Gleeson and Adlard, 2012, *C. pristiophori* Woolcock, 1936, *C. scyliorhinum* Noble, 1948, *C. schulmani* Kovaljova, 1988, and *C. striatellus* Kovaljova, 1988. The new species can be distinguished from *C. pristiophori* by having smaller polar capsules (3.3–4.5 *vs.* 5.0–6.0). Furthermore, *C. atlantoraji* n. sp. has a smaller number of elevated surface ridges (3, rarely 4 ridges) than *C. kuhlii* (4, rarely 5 ridges), *C. lesteri* (4, rarely 5 ridges), *C. leydigi* (4–5 ridges), *C. scyliorhinum* (5 ridges), *C. schulmani* (13 ridges), and *C. striatellus* (8 ridges). Moreover, the new species has very long caudal filamentous projections (3 or 4 times of the myxospore length), while all the above-mentioned species have much shorter ones. Indeed, in *C. scyliorhinum*, projections are as long as its myxospore length, whereas they are shorter than myxospore length in *C. schulmani*, *C. striatellus*, *C. kuhlii*, *C. lesteri*, and *C. mingazzinii*. Genetic differences among the elasmobranch-infecting *Chloromyxum* species that were genetically characterized (SSU rDNA) are shown in Table 1.

**Table 1.** Uncorrected-*p* distance with percentages of sequence similarities (above diagonal) and the number of nucleotide substitutions (below diagonal) among the 14 sequences of *Chloromyxum* spp. included in this study, based on a 1650 bp alignment of the SSU DNA.

	1	2	3	4	5	6	7	8	9	10	11	12	13	14
1 *Chloromyxum zearaji* n. sp. MG652632		99.25	96.02	93.31	93.75	93.79	95.03	94.78	96.22	94.53	94.63	93.50	93.91	90.58
2 *C. atlantoraji* n. sp. MG652633	12		96.03	93.55	93.45	93.55	94.60	94.42	95.87	94.40	94.42	93.06	93.54	90.52
3 *C. riorajum* FJ624481	64	64		99.88	93.13	92.80	94.52	94.20	95.42	93.95	94.19	92.90	93.10	89.93
4 *C. riorajum* MG652631 *	55	53	1		88.73	88.44	90.00	89.35	92.02	89.55	89.81	87.87	88.92	82.40
5 *C. leydigi* AY604199	101	106	111	93		96.18	97.11	97.17	98.80	96.68	96.99	95.97	97.20	90.91
6 *C. clavatum* JQ793641	100	104	116	95	62		96.71	96.40	97.24	96.29	95.65	94.90	95.83	90.80
7 *C. squali* JN130381	68	74	75	58	40	45		96.63	97.22	95.91	95.76	94.83	97.00	90.75
8 *C. mingazzinii* JN130379	71	76	79	61	39	49	46		97.72	97.86	96.33	95.26	97.06	90.42
9 *Chloromyxum* sp. ex *S*. *acanthias* JN130384	52	57	63	47	17	38	38	31		97.08	96.88	96.01	98.55	91.23
10 *C. lesteri* JN130377	75	77	83	61	46	51	56	29	40		96.28	94.90	96.72	90.86
11 *C. kuhlii* JN130375	74	77	80	60	42	60	58	50	43	51		94.01	96.51	90.80
12 *C. hemiscyllii* JN130374	88	94	96	70	55	69	70	64	54	69	81		95.64	90.15
13 *C. leydigi* DQ377710	98	104	111	91	45	67	41	40	20	45	48	59		90.51
14 *C. myliobati* JN130380	130	131	139	104	126	127	127	131	121	126	127	134	131	

* Partial sequence (821 nt from 5′ end).

### *Chloromyxum zearaji* n. sp.

urn:lsid:zoobank.org:act:935ED1FB-732D-43A2-82F2-19781FF682EF

*Type host:* yellownose skate *Zearaja chilensis* (Guichenot) (Rajiformes: Rajidae)

*Type locality:* off the coast of Buenos Aires Province (34°–42°S; 53°–62°W), Argentina.

*Localization of sporogonic stages*: plasmodia and myxospores freely floating in the bile.

*Prevalence of infection:* 45.5% (10 infected in 22 examined fishes).

*Type material:* Syntype MLP-Oi 4151 (coverslip with gold-palladium coated sample for SEM); voucher MLP-ZI-RG 418 (bile containing myxospores in absolute ethanol) deposited in the Other Invertebrates Collection and Genetic Resources Collection, respectively, Museo de La Plata, La Plata, Argentina.

*DNA sequences.* SSU rDNA sequence available in GenBank under the accession number MG652632.

*Etymology:* specific epithet refers to the generic name of its host.

**Figure 3.**
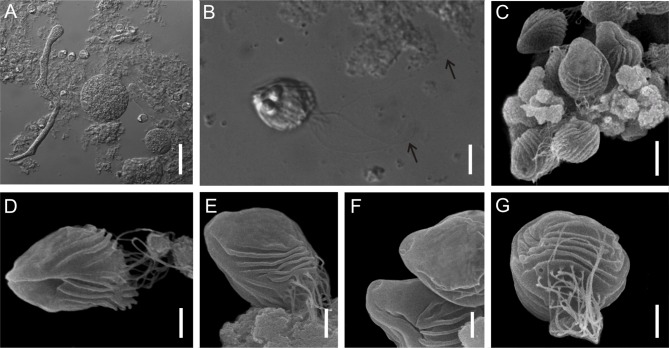
*Chloromyxum zearaji* n. sp. from the gall bladder of the yellownose skate, *Zearaja chilensis* (Guichenot) (Rajidae, Rajiformes). (A) Polymorphic plasmodia and myxospores free in the bile; (B) Mature myxospore showing caudal filamentous projections (arrow), sutural view (DIC micrographs); (C–E) Mature myxospores; (F) Detail of the anterior apex of two myxospores showing the mild depression area for polar filaments extrusion; (G) Mature myxospore, caudal view. Scale bars: A = 40 μm; B, C = 5 μm; D, E, G = 2 μm; Fig. F = 1 μm.

**Figure 4.**
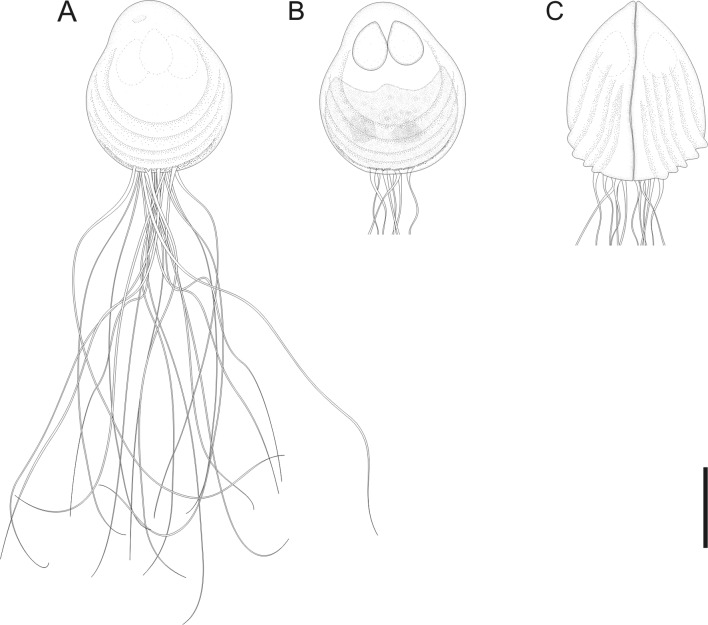
Schematic drawing a myxospore of *Chloromyxum zearaji* n. sp. from the gall bladder of the yellownose skate, *Zearaja chilensis* (Guichenot) (Rajidae, Rajiformes). (A) Valvular view of a mature myxospore. (B) Valvular view of a mature spore showing a single sporoplasm bearing two nuclei. (C) Sutural view of a mature myxospore, caudal filamentous projections not fully drawn. Scale bars: A, B = 5 μm.

*Description* (Figs. 3 and 4)

*Sporogonic stages*: plasmodia polysporic, highly polymorphic; spherical, oval or very irregular. Dimensions varying greatly as a result of polymorphic nature of these structures.

*Myxospores*: mature myxospores ovoid with pointed anterior apex; 11.6 ± 0.4 (10.8–12.4, *n* = 23) long in sutural view, 9.6 ± 0.5 (9.0–10.4, *n* = 23) wide and 9.8 ± 0.4 (9.0–10.6, *n* = 23) thick. Myxospore wall composed of two equally sized valves adhering to each other along a slightly sinuous suture line. Each valve with 5–6 elevated surface ridges parallel to the suture line and nearly parallel to each other and not reaching the apical pole. A bundle of very long caudal filamentous projections, 3 times longer than the myxospore length, originating from the basal portion of shell valves. Four anteriorly pointed, pyriform, equally-sized polar capsules, 3.9 ± 0.3 (3.1–4.6, *n* = 23) in length and 2.7 ± 0.3 (2.1–3.2, *n* = 23) in width. Number of polar filaments coils not determined. Extrusion polar filament area located in a mild depression near anterior apex of each shell valve. Single sporoplasm irregular in shape, bearing two nuclei randomly positioned in its matrix.

#### Taxonomic affinities

Among the *Chloromyxum sensu stricto* representatives and taking into account myxospore dimensions (considering the minimum and maximum values of myxospore length, thickness and width provided in the literature), *C. zearaji* n. sp. most closely resembles *C. hemiscyllii* Gleeson and Adlard, 2012, *C. kuhlii* Gleeson and Adlard, 2012, *C. leydigi* Mingazzini, 1890, *C. myliobati* Gleeson and Adlard, 2012, *C. ovatum* Jameson, 1929, *C. scyliorhinum* Noble, 1948, *Chloromyxum* sp. ex *S. acanthias*, and *C. squali* Gleeson and Adlard, 2012. However, *C. zearaji* n. sp. has a larger number of elevated surface ridges (5, rarely 6 ridges) compared to *C. hemiscyllii* (no visible surface ridges), *C. kuhlii* (4, rarely 5 ridges), *C. myliobati* (3–4 ridges), and *C. ovatum* (3–4 ridges). In addition, the new species has very long caudal filamentous projections (3 times the myxospore length) while in *C. hemiscyllii*, *C. kuhlii*, *C. myliobati*, *C. ovatum*, *C. scyliorhinum*, *Chloromyxum* sp. ex *S. acanthias* and *C. squali* projections are either equal to or shorter than myxospore length. Moreover, the new species can be distinguished from *C. scyliorhinum* by having slightly larger (3.1–4.6 *vs.* 3) and wider (2.1–3.2 *vs.* 1.9) polar capsules.

*Chloromyxum zearaji* can be distinguished from its sympatric congener *C. atlantoraji* n. sp. by myxospore dimensions; being myxospores of *C. zearaji* larger (10.8–12.4 *vs.* 9.7–11.4), thicker (9.0–10.4 *vs.* 7.7–9.4) and wider (9.0–10.4 *vs.* 8.0–9.4), and by having larger number of elevated surface ridges (5–6 ridges *vs.* 3–4 ridges). Genetic differences among the elasmobranch-infecting *Chloromyxum* species that were genetically characterized (SSU rDNA) are shown in Table 1.

### *Chloromyxum riorajum* Azevedo, Casal, Garcia, Matos, Teles-Grilo and Matos, 2009

*Host:* Rio skate *Rioraja agassizii* (Müller and Henle) (Rajiformes: Arhynchobatidae)

*Locality:* off the coast of Buenos Aires Province (34°–42°S; 53°–62°W), Argentina.

*Localization of sporogonic stages*: plasmodia and myxospores floating in the bile.

*Prevalence of infection:* 83.3% (15 infected in 18 examined fishes).

*Material deposited*: vouchers MLP-Oi 4952 (coverslip with gold-palladium coated sample for SEM) and MLP-ZI-RG 419 (bile containing myxospores in absolute ethanol) deposited in the Other Invertebrates Collection and Genetic Resources Collection, respectively, Museo de La Plata, La Plata, Argentina.

*DNA sequences.* SSU rDNA sequence available in GenBank under the accession number MG652631.

*Remarks* (Fig. 5)

**Figure 5.**
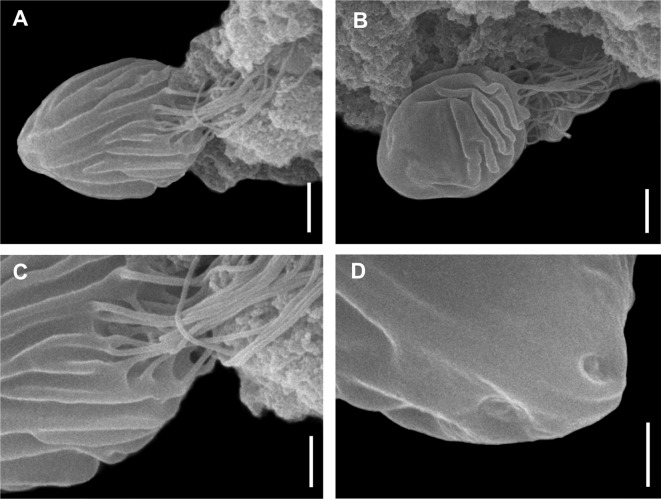
*Chloromyxum riorajum* Azevedo, Casal, García, Matos, Teles-Grilo and Matos, 2009 from the gall bladder of the Rio skate, *Rioraja agassizii* (Müller and Henle) (Arhynchobatidae, Rajiformes); scanning electron micrographs. (A) Mature myxospore, sutural view; (B) Mature myxospore, valvular view; (C) Detail of the caudal bundle of filaments; (D) Detail of the anterior apex showing the mild depression area for polar filaments extrusion. Scale bars: A, B = 2 μm; C, D = 1 μm.

Myxospore dimensions obtained in the present work agree with those reported in the original descriptions of *C. riorajum* [8]. Moreover, the SSU rDNA partial sequence of *C. riorajum* obtained from *R. agassizii* captured off the coast of Buenos Aires Province, Argentina was almost identical (0.1% sequence divergence) with the published partial sequence of *C. riorajum* (GenBank accession no: FJ624481) obtained from the same host from the South Atlantic coast of Brazil.

### Morphometric comparison of myxospores

**Figure 6.**
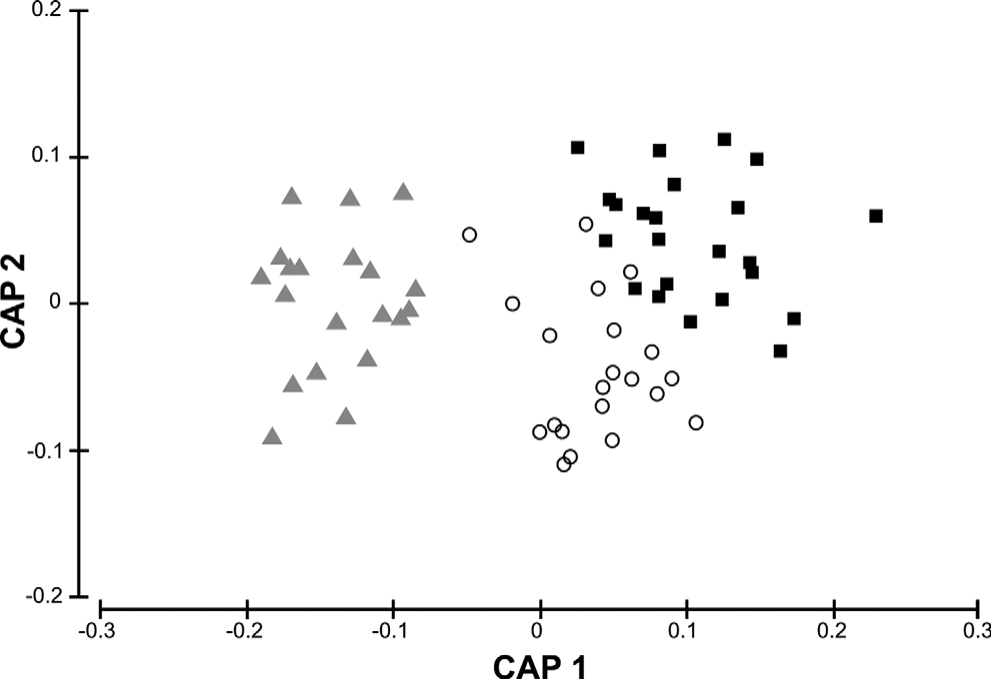
Canonical analysis of principal coordinates (CAP) based on Euclidean distance showing the axes that best discriminate morphometric differences among *Chloromyxum* myxospores from different skate species (∆ the yellownose skate, *Zearaja chilensis* (Guichenot) (Rajidae, Rajiformes), ^○^ the spotback skate, *Atlantoraja castelnaui* (Miranda Ribeiro) (Arhynchobatidae, Rajiformes) and ■ the Rio skate, *Rioraja agassizii* (Müller and Henle) (Arhynchobatidae, Rajiformes)) obtained from commercial trawlers in waters off Buenos Aires Province (38°–42°S), Argentina during 2014.

The CAP analysis showed significant morphometric differences between *Chloromyxum* myxospores from the three skate species. The selected orthonormal PCO axes (*m* = 5), described 100% of the variation in the data cloud, with a high percentage of correct allocations (92.76%) (Fig. 6).

Results of PERMANOVA analyses showed significant morphometric variability among myxospores from the three host species (Pseudo-*F*_2,66_ = 59.708, *P*(perm) = 0.0001). Pairwise tests between samples showed that there were significant differences for all comparisons (for all pairwise comparisons *P*(perm) = 0.0001). None of these differences can be attributed to differences in multivariate dispersions of myxospore measurements in terms of their deviations from centroids, since PERMDISP results were not significant (*F*_2,66_ = 1.22, *P*(perm) > 0.05).

### Molecular characterization and phylogeny

The assemblages of amplified sequences resulted in consensus of almost complete SSU rDNA sequences of *C. atlantoraji* n. sp. (1804 bp) and *C. zearaji* n. sp. (1828 bp) and of partial sequence of *C. riorajum* (821 bp). These sequences were aligned with 11 SSU rDNA of the species that form the *Chloromyxum sensu stricto* clade and two sequences selected as outgroup (Table 2). The resulting alignment consisted of 1650 positions.

**Table 2.** *Chloromyxum* species included in this study, ordered alphabetically.

Species	GenBank accession no.	Length of sequence	Host	Host Family: Order	Locality	Reference
*Chloromyxum atlantoraji* n. sp.	MG652633	1804	*Atlantoraja castelnaui* (Miranda Ribeiro)	Arhynchobatidae: Rajiformes	South Atlantic, coast of Argentina	Present study
*C. clavatum*	JQ793641	1844	*Raja clavata* Linnaeus	Rajidae: Rajiformes	Atlantic coast of Portugal	[72]
*C. hemiscyllii*	JN130374	1355	*Hemiscyllium ocellatum* (Bonnaterre)	Hemiscylliidae: Orectolobiformes	Australian waters	[35]
*C. kuhlii*	JN130375	1386	*Neotrygon kuhlii* (Müller and Henle)	Dasyatidae: Myliobatiformes	Australian waters	[35]
*C. lesteri*	JN130377	1379	*Cephaloscyllium laticeps* (Duméril)	Scyliorhinidae: Carcharhiniformes	Australian waters	[35]
*C. leydigi*	DQ377710	1866	*Centroscymnus coelolepis* Barbosa du Bocage and de Brito Capello	Somniosidae: Squaliformes	North Atlantic, coast of USA	[28]
*C. leydigi*	AY604199	1868	*Torpedo marmorata* Risso	Torpedinidae: Torpediniformes	Mediterranean Sea, coast of Croatia	[30]
*C. mingazzinii*	JN130379	1366	*Pristiophorus nudipinnis* Günther	Pristiophoridae: Pristiophoriformes	Australian waters	[35]
*C. myliobati*	JN130380	1400	*Myliobatis australis* Macleay	Myliobatidae: Myliobatiformes	Australian waters	[35]
*C. riorajum*	MG652631	821	*Rioraja agassizii* (Müller and Henle)	Arhynchobatidae: Rajiformes	South Atlantic, coast of Argentina	Present study
*C. riorajum*	FJ624481	1807	*Rioraja agassizii* (Müller and Henle)	Arhynchobatidae: Rajiformes	South Atlantic, coast of Brazil	[8]
*C. squali*	JN130381	1381	*Squalus acanthias* Linnaeus	Squalidae: Squaliformes	Australian waters	[35]
*C. zearaji* n. sp.	MG652632	1828	*Zearaja chilensis* (Guichenot)	Rajidae: Rajiformes	South Atlantic, coast of Argentina	Present study
*C.* sp. ex *S. acanthias*	JN130384	1399	*Squalus acanthias* Linnaeus	Squalidae: Squaliformes	Australian waters	[35]

**Figure 7.**
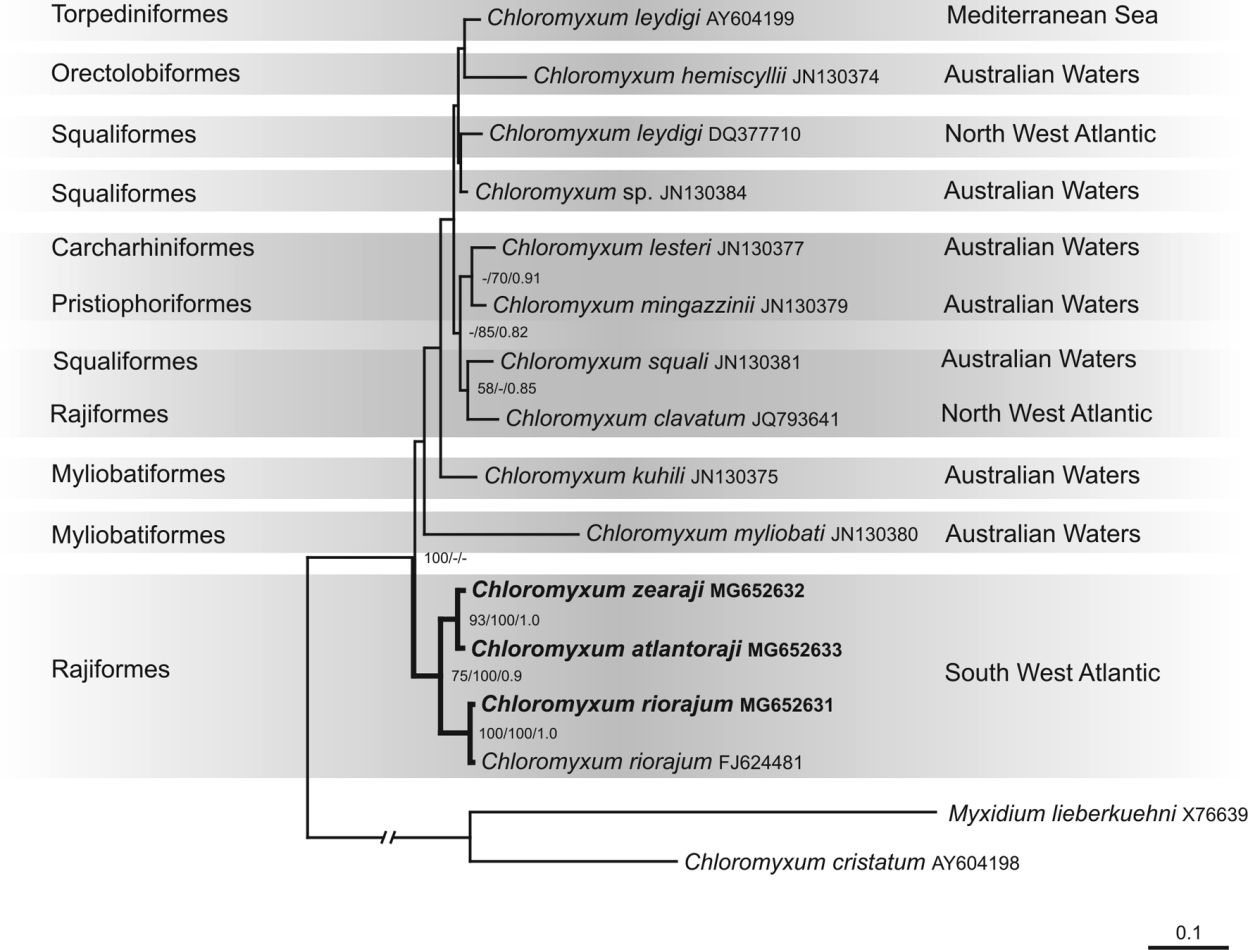
The maximum likelihood tree showing the phylogenetic position of *Chloromyxum* species with new sequence data within *Chloromyxum s.s*. clade. *Chloromyxum cristatum* Léger, 1906 (GenBank accession no: AY604198) and *Myxidium lieberkuehni* Bütschli, 1882 (GenBank accession no: X76639) were set as the outgroup, their common branch shortened to 50% of the original length. Nodal supports are indicated for ML (1000 replicates, only bootstrap values greater than 50% are shown), MP (1000 replicates only bootstrap values greater than 50% are shown) and BI (only posterior probabilities greater than 0.8 are shown). Bold branches lead to nodes highly supported (>70% for ML and MP, and >0.8 for BI) and dashes indicate bootstrap values <50 or node not present in the MP or BI tree. Newly sequenced taxa in bold. Species names are supplemented with corresponding GenBank accession numbers. Host taxonomic order and its geographic distribution are stated at the left and right sides of the ML tree respectively. The number of changes per site is given by the scale bar.

Maximum likelihood analyses of the SSU rDNA sequences revealed that sequences of *Chloromyxum* spp. from Rajiformes of the Atlantic coast of Argentina and the sequence of *C. riorajum* (GenBank accession no: FJ624481) from Brazilian waters group together, and form a sister clade to the rest of the *Chloromyxum sensu stricto* spp. (Fig. 7). MP analysis showed very similar topology differing from ML in basal position of *C*. *myliobati* to all chloromyxid spp. (data not shown). BI analysis did not resolve the position of *C*. *myliobati* and resulted in the polytomy of this species and the clade with chloromyxids from the Atlantic coast of Argentina and the sequence of *C. riorajum* (FJ624481) and the clade containing the rest of *Chloromyxum* spp. The trees are typical for their overall low nodal support except for the highly supported clade (maximum nodal support in all analysis) of the *Chloromyxum* spp. from the Atlantic coast of Argentina and the sequence of *C. riorajum* (FJ624481).

The analyses of interspecies SSU rDNA sequences distances based on a 1650 bp alignment revealed a minimum sequence dissimilarity between *C. atlantoraji* n. sp. and *C. zearaji* n. sp. (12 nt changes, 0.75% dissimilarity); whereas the greatest genetic distance was obtained between *C. riorajum* (FJ624481) and *C. myliobati* (JN130380) (139 nt changes, 10.07% dissimilarity) (Table 1). Within the only well-defined and statistically supported monophyletic subclade embracing *Chloromyxum* species from Rajiformes from the South West Atlantic Ocean (Fig. 7), the similarity among species with almost complete sequences ranged from 96.02% to 99.25%, corresponding to a minimum of 12 and a maximum of 64 nucleotide substitutions (Table 1). Comparison of the partial sequence of *C*. *riorajum* from *Rioraja agassizii* from the coast of Buenos Aires province with *C*. *riorajum* from the same host from the South Atlantic coast of Brazil revealed one nucleotide difference (99.88% similarity).

## Discussion

The examination of 217 gall bladders from seven skate species, representatives of two families of Rajiformes that inhabit waters off the Buenos Aires Province in the Argentine sea, resulted in the finding of three species of *Chloromyxum* in three host species. Their morphological and genetic (SSU rDNA) characterization showed that they represent two new and one previously described species.

Traditionally, the taxonomy of the Myxozoa has been largely based on the morphology and morphometrics of the myxospore stages, resulting in many taxonomic ambiguities [57] due to the simplicity of the microscopic myxospores, to the small number of measurable characters and to their considerable morphological plasticity that result in convergent morphotypes [29]. These features pose difficulties for species identification and discrimination when myxospore attributes are analyzed individually. Nevertheless, the use of multivariate procedures on a set of myxospore measurements (e.g. CAP and PERMANOVA) clearly differentiated the three *Chloromyxum* species considered here, even though some characters displayed overlapping ranges, demonstrating the utility of these methods for specific discrimination purposes.

In any case, the combination of morphological, genetic, and biological information (e.g. geographic locality, host species, tissue tropism, etc.) enables comprehensive informative taxonomic discrimination at the species level. Therefore, the erection of new species should always be accompanied by the application of comprehensive morphological and biological data, as well as molecular analyses to warrant an accurate description [42, 50, 57].

The first taxonomic studies of elasmobranch-infecting *Chloromyxum*, that pre-date the molecular era, were solely based on morphological comparison of myxospores using LM observations [7, 34, 44, 52, 54, 69]. The lack of implementation of ultrastructural (TEM and SEM) and molecular approaches led to poor descriptions that provided few reliable features for taxonomic comparison and led to erroneous identifications. This is also the case of the five *Chloromyxum* species thus far registered in the Atlantic coast of Argentina by Kuznetsova [54], (i) *C. ovatum* from *Mustelus californicum* [valid as *Mustelus californicus* Gill] (Triakidae), *Squalus sucklii* [valid as *Squalus suckleyi* (Girard)] (Squalidae), *Galeorchinus zyopterus* [valid as *Galeorhinus galeus* (Linnaeus)] (Triakidae), *Tetraonarce californica* [valid as *Tetronarce californica* (Ayres)] (Torpedinidae) and *Prionace glauca* (Linnaeus) (Carcharhinidae); (ii) *C. liae* from *P. glauca*; (iii) *C. transversocostatum* from *Squalus fernandinus* [valid as *Squalus acanthias* Linnaeus] (Squalidae); (iv) *C. multicostatum* from *Squatina squatina* (Linnaeus) (Squatinidae), and (v) *C. parvicostatum* from *Raja brachyurops* [valid as *Bathyraja brachyurops* (Fowler)] (Arhynchobatidae) and *Raja magellanica* [valid as *Bathyraja magellanica* (Philippi)] (Arhynchobatidae).

There are several drawbacks within Kuznetsova’s [54] work. Firstly, the species descriptions certainly do not meet current requirements in order to accomplish an accurate taxonomic description and could hinder correct designation of existing as well as new species. Secondly, some of the host species of *Chloromyxum* spp. reported from the Argentine Sea are not distributed in this region. Indeed, *M. californicus* and *S. suckleyi* are restricted to the North Pacific Ocean; *T. californica* is limited to the Eastern Pacific Ocean and Japan coast, and *S. squatina* only inhabits waters of the North Atlantic Ocean [33]. Myxozoans are mostly host-specific parasites in their fish hosts; with individual parasites species developing in only one host species or in a limited number of closely related ones [65, 66], which is why host identity has become an important character in myxozoan taxonomy and their correct identification is mandatory. Lastly, it should be mentioned that *C. ovatum* is now considered an assemblage of several species [35, 72]. Indeed, *C. ovatum* has been recorded from the gall bladder of 9 species of sharks and rays from the Pacific coast of the USA, the Atlantic coast of Argentina and the Atlantic coast of Africa [44, 52, 54], and having such low host specificity and broad geographical area is rather unusual for myxozoans and casts doubts on the actual identity of its components, an issue requiring resolution.

Due to the poorly detailed description available for *C. multicostatum*, that precludes its discrimination from congeners, as well as to the misidentification of the only host reported, this taxon is relegated to the status of a *species inquirenda*. Indeed, three species of elasmobranch genus *Squatina* inhabit the Atlantic coast of Argentina, *S. argentina* (Marini), *S. guggenheim* Marini and *S. occulta* Vooren and Da Silva [60], therefore it is not possible to identify which species was misidentified as *S. squatina* by Kuznetsova [54].

Certainly, future implementation of molecular approaches and morphological revision of *Chloromyxum* species reported in chondrichthyan from the Argentine Sea by Kuznetsova [54] will undoubtedly lead to reassessments (including redescriptions) of these taxa and further resolution of possible cryptic species.

The advent of PCR methods and readily accessible DNA sequencing technologies have advanced our general understanding of myxozoan phylogenetic relationships and taxonomy. In particular, the SSU rDNA sequence has become an integral part of myxozoan species descriptions and phylogenetic studies. Despite the increasing number of available myxozoan SSU rDNA records in GenBank, the proportion of sequenced *Chloromyxum* species infecting the gall bladder of marine elasmobranch remains low. Indeed, the GenBank database provides information for only 10 (out of 25) elasmobranch-infecting *Chloromyxum* species (see Table 2).

Phylogenetic analyses have shown that primary myxosporean division is according to their host environment (either freshwater or marine realm) [28], with a third main group being the *Sphaerospora sensu stricto* clade from mixed environments [14, 46]. Clustering within these lineages appears to be related to the site of sporulation in the intermediate vertebrate host (coelozoic infecting the gall bladder, urinary bladder and kidney tubules or histozoic infecting muscles or other tissues) [41]. The *Chloromyxum s.s* clade uniting the marine gall bladder-infecting species is placed basally to the members of the freshwater myxosporean lineage [8, 13, 30, 35, 72], suggesting that the common ancestor of the freshwater myxosporean lineage was a parasite of the elasmobranchs [51]. In any event, as expected due to myxospore morphotype, host identity (elasmobranchs) and tissue infected (gall bladder), the three *Chloromyxum* species sequenced in this study occupy a position within the well-supported *Chloromyxum s.s* clade.

Although not without limitations and weaknesses (e.g. Long-branch attraction artefact [15]), the SSU rRNA gene has repeatedly proved to be sufficiently informative to estimate the phylogenetic relationships among myxozoans at different taxonomic levels [31]. However, single-gene analyses sometimes do not provide sufficient resolution for some nodes or sometimes give conflicting results. In fact, the species-level SSU rDNA phylogeny presented in this work could not be completely resolved, containing both well-supported and low-supported nodes. Indeed, while *Chloromyxum* parasites of Rajiformes from the South Atlantic Ocean (*C. atlantoraji* n. sp., *C. zearaji* n. sp. and *C. riorajum*) constituted a well-established monophyletic subclade with strong support for the three analytical methods used, nodes grouping the other *Chloromyxum* species, independently of the host taxonomic identities (Carchariniformes, Myliobatiformes, Orectolobiformes, Pristiophoriformes, Rajiformes, Squaliformes and Torpediniformes) and/or host range geographic distribution (Atlantic coast of Portugal, Atlantic coast of the USA, Australian waters or Mediterranean Sea), were poorly resolved or unresolved and, therefore, unclear.

The lack of resolution observed in the single-gene tree reconstruction presented in this work could be due to either artefacts of the inferences processes (analytical factors [15]), insufficient taxon sampling or may be biologically real. In an attempt to allow more accurate reconstructions with the current data set, different methodological approaches were implemented (results not shown), including variation in the stringency of removal of ambiguous characters of the input alignment and/or selection of different taxa as outgroup (i) taxa from a distant clade e.g. *Kudoa carcharhini* GU324970 + *Ceratomyxa carcharhini* JF911815; (ii) a single taxon from the sister freshwater hepatic biliary clade e.g. *Sphaeromyxa balbiani* KF 135225; (iii) multiple taxa within the sister freshwater hepatic biliary clade e.g. *S. balbiani* KF135225 + *Myxidium coryphaenoideum* DQ377697 + *Zschokkela nova* DQ377690).In fact, none of these attempts overcame the lack of resolution and node instability observed.

Indeed, the difficulty in the phylogenetic reconstruction faced in the present work could be ascribed to the low amount of phylogenetic signal shown by the SSU rDNA molecular marker within the closely related *Chloromyxum s.s.* –e.g. speciation events closely spaced in time-. Acknowledging that it might not always be possible to obtain a fully-resolved and well-supported species tree [10, 43], further improvements of *Chloromyxum s.s.* phylogeny could be achieved by the inclusion of data from other loci – e.g. LSU rDNA and/or EF-2 gene [13, 29]- or by increase taxon sampling in order to strengthen the phylogenetic signal.

A phylogeny of a group of organisms has several determinants in the historical context for biogeography and ecology. Particularly, phylogenies of parasite taxa are structured and historically constrained by genealogical and ecological associations with their host (or hosts for parasites with complex life cycles) within the context of frameworks and hypotheses for co-evolution (encompassing co-speciation and co-adaptation) and historical biogeography across varying temporal (evolutionary to ecological time) and spatial scales (local, regional to global) [38]. Though elucidating the processes and contingencies that structured the phylogenetic relationships among *Chloromyxum s.s.* species is beyond the scope of the present work, some clear macroevolutionary patterns were depicted from the tree topology inferred in synopsis with biological and ecological aspects of *Chloromyxum* species and their hosts.

Analysing data in the context of host identity-based structure, one of the most prominent results is the partial incongruence and inconsistence of phylogenies for the genus *Chloromyxum* and their elasmobranch intermediate hosts, with closely related hosts not harbouring closely related parasites. At least for those species parasitizing Rajiformes, the only well supported sublineage from the South West Atlantic Ocean, clearly do not encompass the other *Chloromyxum* species from Rajiformes included in the analyses (*C. clavatum* from the Atlantic coast of Portugal) that clustered separately. Of course, this pattern is not surprising as departures from strict co-speciation are frequently observed. Such lack of complete congruence could be a consequence of many macroevolutionary events from deep evolutionary to shallow ecological timeframes (e.g. host switching, sorting or extinction, geographical dispersal, duplication or intrahost speciation, inertia or lack of parasite speciation, colonization, etc.) [9, 17, 39, 68].

In phylogeny-based approaches, hierarchical order over spatial and temporal scales constrains the range of explanations for understanding patterns that have driven diversification in evolutionary and biogeographic history [37, 38]. As a generality in host-parasite assemblages, geographic scale may be linked to the relative age of the host-parasite association and to the duration of their co-evolutionary history [38]. In this sense, the global distribution of the members of the genus *Chloromyxum* may be indicative of an old association with their specific host group (elasmobranchs), as it is proven for other groups of parasites [16, 17, 21, 40]. The same is true at a smaller geographic scale for chloromyxids found infecting south Atlantic skates, whose association could not pre-date Early Cretaceous times when the South Atlantic Ocean originated [23]. Estimating divergence time from molecular sequence data for *Chloromyxum* species and their hosts could further contribute to address the relative age of these associations.

It is noteworthy that the only well-supported subclade of *Chloromyxum* species is composed of sympatric members. Therefore, it is reasonable to infer that the cladogenesis of these species may be the result of a colonization event followed by local isolation and diversification, a process requiring further analyses considering the geographical and temporal bounds of this particular ecological zone. Globally, the Southwestern Atlantic Ocean has one of the highest degrees of endemism in skates [24, 32] with the genera *Rioraja* (Whitley) and *Atlantoraja* (Menni) being endemic [24, 32]. On the other hand, *Zearaja* (Whitley) is a cosmopolitan genus; however, *Z. chilensis* is restricted to the South American shelf, inhabiting Atlantic and Pacific waters [61]. The isolation of this habitat maintained over evolutionary time may have contributed to increase the diversity of endemic hosts and parasites through coevolutionary processes. In fact, it is proven that areas of high host endemism are also areas of parasite endemism [67]. Molecular analyses, including of new *Chloromyxum* species from elasmobranchs belonging to different families and orders from the South Atlantic Ocean, will allow us to assess the evolution of these geographical lineages.

## References

[1] Anderson MJ. 2001 A new method for non-parametric multivariate analysis of variance. Austral Ecology, 26, 32–46.

[2] Anderson MJ, Robinson J. 2003 Generalized discriminant analysis based on distances. Australian & New Zealand Journal of Statistics, 45, 301–318.

[3] Anderson MJ, Willis TJ. 2003 Canonical analysis of principal coordinates: a useful method of constrained ordination for ecology. Ecology, 84, 511–525.

[4] Anderson MJ, Gorley RN, Clarke KR. 2008 PERMANOVA + for PRIMER: Guide to Software and Statistical Methods. PRIMER-E Ltd.: Plymouth.

[5] Asahida T, Kobayashi T, Saitoh K, Nakayama I. 1996 Tissue preservation and total DNA extraction from fish stored at ambient temperature using buffers containing high concentration of urea. Fisheries Science, 62, 727–730.

[6] Atkinson SD, Bartošová-Sojková P, Whipps CM, Bartholomew JL. 2015 Approaches for characterising Myxozoan species, in Myxozoan evolution, ecology and development. Okamura B, Gruhl A, Bartholomew JL, Editors. Springer: London p. 111–123.

[7] Awerinzew S. 1913 Ergebnisse der Intersuchungen über parasitische Protozoen der tropischen Region Afrikas. Zoologischer Anzeiger, 42, 151–156.

[8] Azevedo C, Casal G, García P, Matos P, Teles-Grilo L, Matos E. 2009 Ultrastructural and phylogenetic data of *Chloromyxum riorajum* sp. nov. (Myxozoa), a parasite of the stingray *Rioraja agassizii* in Southern Brazil. Diseases of Aquatic Organisms, 85, 41–51. 19593932 10.3354/dao02067

[9] Barker SC. 1991 Evolution of host-parasite associations among species of lice and rock wallabies: coevolution? International Journal for Parasitology, 21, 497–501. 1743847 10.1016/0020-7519(91)90053-a

[10] Barraclough TG, Nee S. 2001 Phylogenetics and speciation. Trends in Ecology & Evolution, 16, 391–399. 11403872 10.1016/s0169-5347(01)02161-9

[11] Barta JR, Martin DS, Liberator PA, Dashkevicz M, Anderson JW, Feighner SD, Elbrecht A, Perkins-Barrow A, Jenkins MC, Danforth HD, Ruff MD, Profous-Juchelka H. 1997 Phylogenetic relationship among eight *Eimeria* species infecting domestic fowl inferred using complete small subunit ribosomal DNA sequences. Journal of Parasitology, 83, 262–271. 9105308

[12] Bartošová P, Fiala I. 2011 Molecular evidence for the existence of cryptic species assemblages of several myxosporeans (Myxozoa). Parasitology Research, 108, 573–583. 20938686 10.1007/s00436-010-2100-y

[13] Bartošová P, Fiala I, Hypša V. 2009 Concatened SSU and LSU rDNA data confirm the main evolutionary trends within myxosporeans (Myxozoa: Myxozporea) and provide an effective tool for their molecular phylogenetics. Molecular Phylogenetics and Evolution, 53, 81–93. 19477283 10.1016/j.ympev.2009.05.018

[14] Bartošová P, Fiala I, Jirků M, Cinková M, Caffara M, Fioravanti ML, Atkinson SD, Bartholomew JL, Holzer AS. 2013 *Sphaerospora sensu stricto*: Taxonomy, diversity and evolution of a unique lineage of myxosporeans (Myxozoa). Molecular Phylogenetics and Evolution, 68, 93–105. 23500334 10.1016/j.ympev.2013.02.026

[15] Bergsten J. 2005 A review of long-branch attraction. Cladistics, 21, 163–193. 10.1111/j.1096-0031.2005.00059.x 34892859

[16] Boeger WA, Kritsky DC. 1997 Coevolution of the Monogenoidea (Platyhelminthes) based on a revised hypothesis of parasite phylogeny. International Journal for Parasitology, 27, 1495–1511. 9467734 10.1016/s0020-7519(97)00140-9

[17] Brooks DR, McLennan DA. 1993 Parascript – Parasites and the language of evolution. Smithsonian Institution Press: Washington, DC.

[18] Clarke KR, Gorley RN. 2015 PRIMER v7: User Manual/Tutorial. PRIMER-E Ltd.: Plymouth.

[19] Clarke KR, Gorley RN, Somerfield PJ, Warwick RM. 2014 Change in marine communities. An approach to statistical analysis and interpretation. PRIMER-E Ltd.: Plymouth.

[20] Colonello JH, Cortés F, Massa AM. 2014 Species richness and reproductive modes of chondrichthyans in relation to temperature and fishing effort in the Southwestern Atlantic Shelf (34–54° S). Fisheries Research, 160, 8–17.

[21] Cribb TH, Bray RA, Littlewood DTJ. 2001 The nature and evolution of the association among digeneans, molluscs and fishes. International Journal for Parasitology, 31, 997–1011. 11406146 10.1016/s0020-7519(01)00204-1

[22] Díaz de Astarloa JM, Mabragaña E, Hanner E, Figueroa DE. 2008 Morphological and molecular evidence for a new species of longnose skate (Rajiformes: Rajidae: *Dipturus*) from Argentinean waters based on DNA barcoding. Zootaxa, 1924, 35–46.

[23] Eagles G. 2007 New angles on south Atlantic opening. Geophysical Journal International, 168, 353–361.

[24] Ebert DA, Compagno LJV. 2007 Biodiversity and systematic of skates (Chondrichthyes: Rajiformes: Rajoidei). Environmental Biology of Fishes, 80, 111–124.

[25] Eiras JC, Lu YS, Gibson DI, Fiala I, Saraiva A, Cruz C, Santos MJ. 2012 Synopsis of the species *Chloromyxum* Mingazinni, 1890 (Myxozoa: Myxosporea: Chloromyxidae). Systematic Parasitology, 83, 203–225. 23065304 10.1007/s11230-012-9380-9

[26] Eschmeyer WN, Fricke R, van der Laan R. 2016 Catalog of fishes: genera, species, references. http://researcharchive.calacademy.org/research/ichthyology/catalog/fishcatmain.asp. Accessed 1 August 2017.

[27] Evdokimova EB. 1977 Myxosporidians of teleost fishes from the Patagonian shelf (The Atlantic Coast of Argentina). Parazitologiya, 11, 166–178. 405648

[28] Fiala I. 2006 The phylogeny of Myxosporea (Myxozoa) based on small subunit ribosomal RNA gene analysis. International Journal for Parasitology, 36, 1521–1534. 16904677 10.1016/j.ijpara.2006.06.016

[29] Fiala I, Bartošová P. 2010 History of myxozoan character evolution on the basis of rDNA and EF-2 data. BMC Evolutionary Biology, 10, 228. 20667097 10.1186/1471-2148-10-228 PMC2927925

[30] Fiala I, Dyková I. 2004 The phylogeny of marine and freshwater species of the genus *Chloromyxum* Mingazzini, 1890 (Myxosporea: Bivalvulida) based on small subunit ribosomal RNA gene sequences. Folia Parasitologica, 51, 211–214. 15357399

[31] Fiala I, Bartošová-Sojková P, Whipps CM. 2015 Classification and phylogenetics of Myxozoa, in Myxozoan evolution, ecology and development. Okamura B, Gruhl A, Bartholomew JL, Editors. Springer: London p. 85–110.

[32] Figueroa DE, Barbini S, Scenna L, Belleggia M, Delpiani G, Spath C. 2013 El endemismo en las rayas de la Zona Común de Pesca Argentino-Uruguaya. Frente Marítimo, 23, 95–104.

[33] Froese R, Pauly D. 2016 FishBase. World Wide Web electronic publication. http://fishbase.org. Accessed 1 August 2017.

[34] Gioia I, Cordeiro NS. 1996 Brazilian myxosporidians’ checklist (Myxozoa). Acta Protozoologica, 35, 137–149.

[35] Gleeson RJ, Adlard RD. 2012 Phylogenetic relationship amongst *Chloromyxum* Mingazzini, 1890 (Myxozoa: Myxosporea), and the description of six novel species from Australian elasmobranchs. Parasitology International, 61, 267–274. 22085584 10.1016/j.parint.2011.10.008

[36] Hallet SL, Diamant A. 2001 Ultrastructure and small-subunit ribosomal DNA sequence of *Henneguya lesteri* n. sp. (Myxosporea), a parasite of sand whiting *Sillago analis* (Sillaginidae) from the coast of Queensland, Australia. Diseases of Aquatic Organisms, 46, 197–212. 11710554 10.3354/dao046197

[37] Hoberg EP, Brooks DR. 2008 A macroevolutionary mosaic: episodic host-switching, geographical colonization and diversification in complex host–parasite systems. Journal of Biogeography, 35, 1533–1550.

[38] Hoberg EP, Klassen GJ. 2002 Revealing the faunal tapestry: co-evolution and historical biogeography of hosts and parasites in marine systems. Parasitology, 124, S3–S22. 12396213 10.1017/s0031182002001841

[39] Hoberg EP, Brooks DR, Siegel-Causey D. 1997 Host–parasite cospeciation: history, principles and prospects, in Host–parasite evolution: General principles and avian models. Clayton DH, Moore J, Editors. Oxford University Press: Oxford p. 212–235.

[40] Hoberg EP, Gardner SL, Campbell RA. 1999 Systematics of the Eucestoda: advances toward a new phylogenetic paradigm, and observations on the early diversification of tapeworms and vertebrates. Systematic Parasitology, 42, 1–12. 10613542 10.1023/a:1006099009495

[41] Holzer AS, Sommerville C, Wootten R. 2004 Molecular relationships and phylogeny in a community of myxosporeans and actinosporeans based on their 18S rDNA sequences. International Journal for Parasitology, 34, 1099–1111. 15380681 10.1016/j.ijpara.2004.06.002

[42] Holzer AS, Wootten R, Sommerville C. 2010 *Zschokkella hildae* Auerbach, 1910: Phylogenetic position, morphology, and location in cultured Atlantic cod. Parasitology International, 59, 133–140. 20026425 10.1016/j.parint.2009.12.004

[43] Humphries EM, Winker K. 2010 Working through polytomies: Auklets revisited. Molecular Phylogenetics and Evolution, 54, 88–96. 19643194 10.1016/j.ympev.2009.07.023

[44] Jameson AP. 1929 Myxosporidia from Californian fishes. Journal of Parasitology, 16, 59–68.

[45] Jaureguizar AJ, Menni RC, Lasta CA, Guerrero RA. 2006 Fish assemblages of the northern Argentine coastal system: spatial patterns and their temporal variations. Fisheries Oceanography, 15, 326–344.

[46] Jirků M, Fiala I, Modrý D. 2007 Tracing the genus *Sphaerospora*: rediscovery, redescription and phylogeny of the *Sphaerospora ranae* (Morelle, 1929) n. comb. (Myxosporea, Sphaerosporidae), with emendation of the genus *Sphaerospora*. Parasitology, 134, 1727–1739. 17651531 10.1017/S0031182007003241

[47] Jirků M, Bartošová P, Kodádková A, Mutschmann F. 2011 Another chloromyxid lineage: molecular phylogeny and redescription of *Chloromyxum careni* from the Asian Horned frog *Megophrys nasuta*. Journal of Eukaryotic Microbiology, 58, 50–59. 21182559 10.1111/j.1550-7408.2010.00521.x

[48] Katoh K, Misawa K, Kuma K, Miyata T. 2002 MAFFT: a novel method for rapid multiple sequence alignment based on fast Fourier transform. Nucleic Acid Research, 30, 3059–3066. 10.1093/nar/gkf436 PMC135756 12136088

[49] Kent ML, Khattra J, Hervio DML, Devlin RH. 1998 Ribosomal DNA sequence analysis of isolates of the PKX myxosporean and their relationship to members of the genus *Sphaerospora*. Journal of Aquatic Animal Health, 10, 12–21.

[50] Kent ML, Andree KB, Bartholomew JL, El-Matbouli M, Desser SS, Devlin RH, Feist SW, Hedrick RP, Hoffmann RW, Khattra J, Hallett SL, Lester RJG, Longshaw M, Palenzeula O, Siddall ME, Xiao C. 2001 Recent advances in our knowledge of the Myxozoa. Journal of Eukaryotic Microbiology, 48, 395–413. 11456316 10.1111/j.1550-7408.2001.tb00173.x

[51] Kodádková A, Bartošová-Sojková P, Holzer AS, Fiala I. 2015 *Bipteria vetusta* n. sp. – an old parasite in an old host: tracing the origin of myxosporean parasitism in vertebrates. International Journal for Parasitology, 45, 269–276. 25659495 10.1016/j.ijpara.2014.12.004

[52] Kovaljova AA. 1988 Myxoporidia of the genus *Chloromyxum* (Cnidospora, Myxosporea) of cartilaginous fishes from the Atlantic coast of Africa. Parazitologiya, 22, 384–388.

[53] Kudo RR. 1919 Studies on Myxosporidia. A synopsis on genera and species of Myxosporidia. Biological Monographs, 5, 1–265.

[54] Kuznetsova IG. 1977 Myxosporidians of Chondrostei from the Patagonian shelf. Parazitologiya, 11, 74–77. 405646

[55] Lom J, Arthur JR. 1989 A guideline for the preparation of species descriptions in Myxosporea. Journal of Fish Diseases, 12, 151–156.

[56] Lom J, Dyková I. 1992 Protozoan Parasites of Fishes. Developments in Aquaculture and Fisheries. Elsevier: Amsterdam.

[57] Lom J, Dyková I. 2006 Myxozoan genera: definition and notes on taxonomy, life-cycle terminology and pathogenic species. Folia Parasitologica, 53, 1–36. 16696428

[58] Lucifora LO, García VB, Menni RC, Worm B. 2012 Spatial patterns in the diversity of sharks, rays, and chimaeras (Chondrichthyes) in the Southwest Atlantic. Biodiversity and Conservation, 21, 407–419.

[59] Menni RC, López HL. 1984 Distributional patterns of Argentine marine fishes. Physis, 42, 71–85.

[60] Menni RC, Lucífora LO. 2007 Condrictios de la Argentina y Uruguay. Lista de Trabajo. ProBiota, FCNyM, UNLP, Serie Técnica-Didactica, La Plata, Argentina, 11, 1–15.

[61] Menni RC, Stehmann MF. 2000 Distribution, environment and biology of batoid fishes off Argentina, Uruguay and Brazil. A review. Revista del Musea Argentino de Ciencias Naturales (Argentina), 2, 69–109.

[62] Menni RC, Rincón G, García ML. 2008 *Discopyge castelloi* sp. nov. (Torpediniformes, Narcinidae), una nueva especie de raya eléctrica del Mar Argentina. Revista del Musea Argentino de Ciencias Naturales (Argentina), 10, 161–171.

[63] Menni RC, Jaureguizar AJ, Stehmann MFW, Lucifora LO. 2010 Marine biodiversity at the community level: zoogeography of sharks, skates, rays and chimaeras in the Southwestern Atlantic. Biodiversity and Conservation, 19, 775–796.

[64] Mingazzini P. 1890 Sullo sviluppo dei Myxosporidi. Bollettino della Società di Naturalisti, 4, 160–164.

[65] Molnár K. 1994 Comments on the host, organ and tissue specificity of fish myxosporeans and on the types of their intrapiscine development. Parasitologica Hungarica, 27, 5–20.

[66] Molnár K, Eszterbauer E. 2015 Specificity of infection sites in vertebrate hosts, in Myxozoan evolution, ecology and development. Okamura B, Gruhl A, Bartholomew JL, Editors. Springer: London p. 295–313.

[67] Morand S, Guégan J-F. 2000 Patterns of endemism in host-parasite associations: lessons from epidemiological models and comparative test. Belgian Journal of Entomology, 2, 135–147.

[68] Paterson AM, Banks J. 2001 Analytical approaches to measuring co-speciation of host and parasites: through the glass darkly. International Journal for Parasitology, 31, 1012–1022. 11406147 10.1016/s0020-7519(01)00199-0

[69] Pinto C. 1928 Mixosporídeos e outros protozoários intestinais observados na América do Sul. Archivos do Instituto Biológico, 1, 101–102.

[70] Rambaut A, Drummond AJ. 2007 Tracer v1.4 http://beast.bio.ed.ac.uk/Tracer

[71] Rocha S, Azevedo C. 2012 Light and electron microscopy applied to the characterization of marine species belonging to the genus Chloromyxum, as a study model for myxosporean parasites, in Current Microscopy Contributions to Advances in Science and Technology, Méndez-Vilas A, Editor.Formatex Research Center: Badajoz, p. 471–477.

[72] Rocha S, Casal G, Al-Quraishy S, Azevedo C. 2013 Morphological and molecular characterization of a new myxozoan species (Myxosporea) infecting the gall bladder of *Raja clavata* (Chondrichthyes), from the Portuguese Atlantic Coast. Journal of Parasitology, 99, 307–317. 22998576 10.1645/GE-3150.1

[73] Rocha S, Casal G, Al-Quraishy S, Azevedo C. 2014 Morphological and ultrastructural redescription of *Chloromyxum leydigi* Mingazzini, 1890 (Myxozoa: Myxosporea), type species of the genus, infecting the gall bladder of the marine cartilaginous fish *Torpedo marmorata* Risso (Chondrichthyes: Torpedinidae), from the Portuguese Atlantic coast. Folia Parasitologica, 61, 1–10. 24684047

[74] Ronquist F, Huelsenbeck JP. 2003 MrBayes 3: Bayesian phylogenetic inference under mixed models. Bioinformatics, 19, 1572–1574. 12912839 10.1093/bioinformatics/btg180

[75] Ruocco NL, Lucifora LO, Díaz de Astarloa JM, Mabragaña EM, Delpiani MS. 2012 Morphology and DNA barcoding reveal a new species of eagle ray from the Southwestern Atlantic: *Myliobatis ridens* sp. nov. (Chondrichthyes: Myliobatiformes: Myliobatidae). Zoological Studies, 51, 863–873.

[76] Schneider CA, Rasband WS, Eliceiri KW. 2012 NIH Image to ImageJ: 25 years of image analysis. Nature Methods, 9, 671–675. 22930834 10.1038/nmeth.2089 PMC5554542

[77] Stamatakis A. 2006 RAxML-VI-HPC: maximum likelihood-based phylogenetic analyses with thousands of taxa and mixed models. Bioinformatics, 22, 2688–2690. 16928733 10.1093/bioinformatics/btl446

[78] Swofford DL. 2003 PAUP*: Phylogenetic Analysis Using Parsimony (*and other methods). Version 4. Sinauer Associates, Sunderland, Massachusetts.

